# Supplementary figures and images for: Patient and guardian perspectives on tissue engineering in microtia reconstruction
Source: PLoS One. 2025 Dec 19;20(12):e0338194. doi: 10.1371/journal.pone.0338194 (PMC12716780; doi:10.1371/journal.pone.0338194)

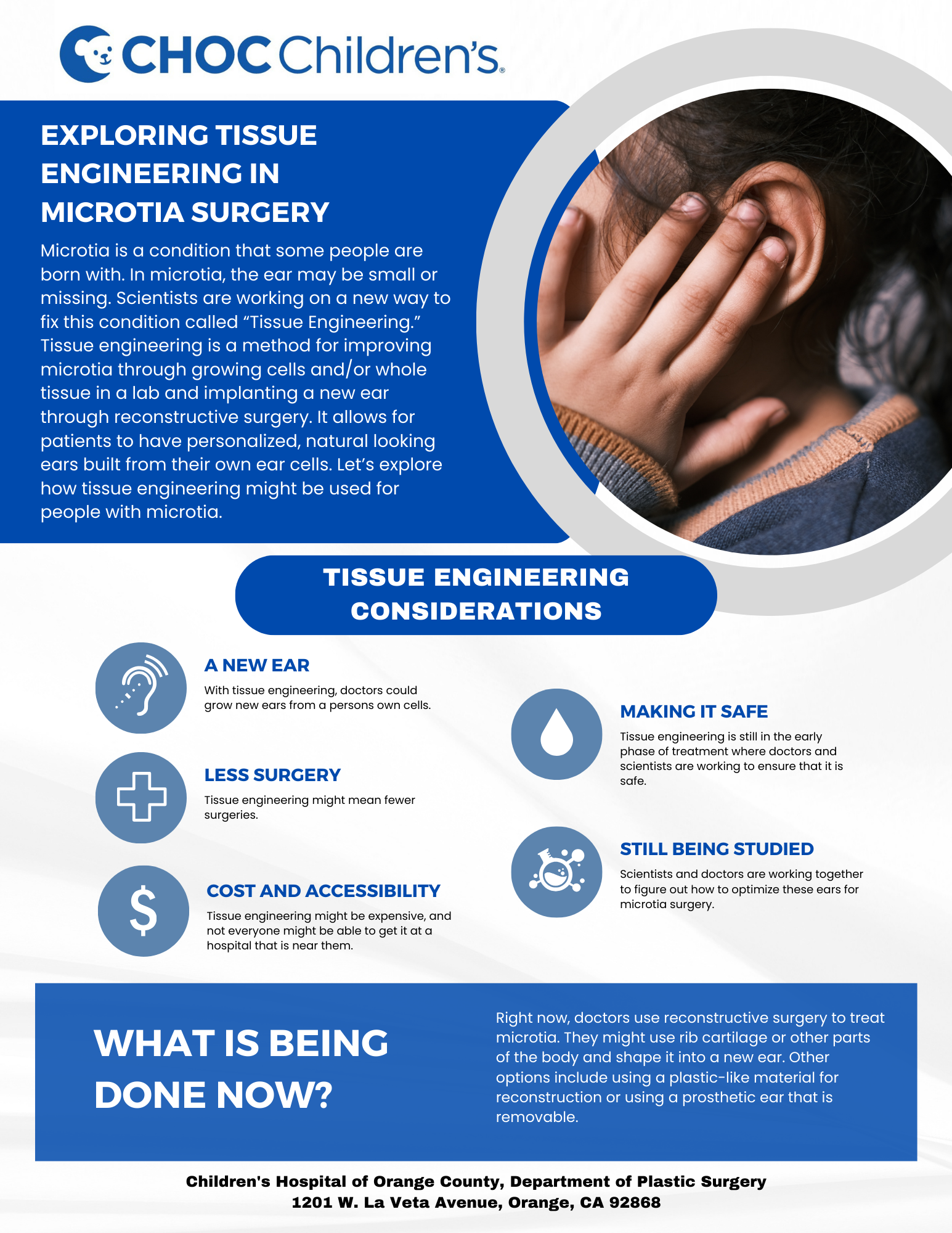

Supplement: S1 Fig — Educational material provided to patients/guardians. (PNG) [file pone.0338194.s001.png]
