# Supplementary material for: Patient and guardian perspectives on tissue engineering in microtia reconstruction
Source: PLoS One. 2025 Dec 19;20(12):e0338194. doi: 10.1371/journal.pone.0338194 (PMC12716780; doi:10.1371/journal.pone.0338194)
Supplement: S2 Fig — Survey provided to parents/guardians. (PDF) [file pone.0338194.s002.pdf]

FOR OFFICE USE:

MRN:

CHOC Children's

CHILDREN'S HOSPITAL OF ORANGE COUNTY
Tissue Engineering Perspectives Survey

PERSONAL INFORMATION:

NAME:

DATE OF BIRTH: DAY / MONTH / YEAR

AGE:

PARENT/GUARDIAN NAME:

PARENT/GUARDIAN RELATIONSHIP:

GENDER OF SURVEYOR:
- Male
- Female
- Transgender Female
- Transgender Male
- Genderqueer
- Additional Gender Category or Other
- Prefer Not to Answer

RACE OF SURVEYOR:
- African American or Black
- American Indian/Alaska Native
- Asian
- Native Hawaiian or Other Pacific Islander
- White
- More than one race
- Other
- Prefer Not to Answer

ETHNICITY OF SURVEYOR:
- Hispanic or Latino
- NOT Hispanic or Latino
- Prefer Not to Answer

INSTRUCTIONS:

We would like to gather your thoughts on ear reconstruction techniques currently used in surgery. Please review the questions below and provide answers based on your beliefs and perspectives as of this visit.

Tissue Engineering is a method where scientists grow cells in a lab to repair or replace body parts. This prevents surgeons from using body parts from someone else or someone's own body.

QUESTIONS:	RATING SCALE:					
	Not at all	No	Somewhat	Yes	Definitely	Decline to Answer
1. How do you feel about new technology being used in surgeries?	○	○	○	○	○	○
2. Have you heard of "Tissue Engineering" before today?	○	○	○	○	○	○
3. Did you know what "Tissue Engineering is before today?"	○	○	○	○	○	○
4. Do you understand how Tissue Engineering could be used to fix body parts?	○	○	○	○	○	○
5. How do you feel about using Tissue Engineering for your child's ear reconstruction?	○	○	○	○	○	○
	My doctor	Medical staff	Friends or Family	Internet	Others	Not applicable
6. Where did you learn about Tissue Enginnering?	○	○	○	○	○	○
7. Do you think Tissue engineering is a good idea for medicine/surgery?	○	○	○	○	○	○
	Not at all	No	Somewhat	Yes	Definitely	Decline to Answer
8. Are you interested in learning more about Tissue Engineering?	○	○	○	○	○	○

QUESTIONS:	Not at all	No	Somewhat	Yes	Definitely	Decline to Answer
1. Have you looked up information about "Microtia" or "Anotia" on your own? **Explanation: Microtia/Anotia are ear abnormalities.**						
2. Do you understand how microtia/anotia occurs when a baby is born?						
3. Do you know the different ways doctors fix microtia/anotia?						
4. Are you interested in Tissue Engineering to fix your child's ear?						
5. Besides surgery in general, do you have any worries about using Tissue Engineering for ear fixing?						
6. Would you choose Tissue Engineering over other ways to fix the ear?						

QUESTIONS:	RATING SCALE:					
	Not at all	No	Somewhat	Yes	Definitely	Decline to Answer
1. Would you believe a doctor who says Tissue Engineering would help fix your child's ear?						
2. Would you trust information that shows Tissue Engineering worked well for other children?						
3. Do you want to see pictures of other kid's results before choosing Tissue Engineering for your child?						

QUESTIONS:	RATING SCALE:					
	Not at all	No	Somewhat	Yes	Definitely	Decline to Answer
1. Would the cost of Tissue Engineering for your child's ear be a big part of your decision to choose Tissue Engineering?						
2. Would there be a cultural reason as to why you may not choose Tissue Engineering?						
3. Would you seek out and travel to a hospital that offers Tissue Engineering?						
