# Supplementary material for: Patient and guardian perspectives on tissue engineering in microtia reconstruction
Source: PLoS One. 2025 Dec 19;20(12):e0338194. doi: 10.1371/journal.pone.0338194 (PMC12716780; doi:10.1371/journal.pone.0338194)
Supplement: Attachment — Submitted filename: comments.docx [file pone.0338194.s005.docx]

The study addresses an important and emerging area in reconstructive surgery—patient and guardian perspectives on tissue engineering for microtia—which has clear clinical and translational relevance. I commend the authors for undertaking this work, engaging directly with patients and families, and presenting novel data in a field where patient-centered perspectives are often underrepresented.

My comments below aim to strengthen the manuscript by clarifying the methods, ensuring appropriate use of statistical analyses, and refining the presentation of results and conclusions. I have organized my feedback into major and minor points, with the goal of providing constructive suggestions to enhance the rigor, clarity, and impact of this valuable contribution.

1. **Small Sample Size vs. Complex Multivariate Models**: The study involves 40 surveys and the use of multivariate/ordinal logistic regressions. Given the likely low events-per-variable ratio, there is a risk of overfitting. Please report the number of predictors per model, consider using simpler models, or apply penalized/Firth logistic regression with accompanying model diagnostics (AIC, goodness-of-fit). If none of these options are feasible, I recommend removing overambitious multivariate claims.
2. **Ambiguity Regarding Timing of Educational Material**: The methods state that an educational document was “distributed… prior to the initiation of the survey,” yet the results indicate participants’ familiarity was assessed “before receiving supplemental educational material.” This is contradictory; please clarify whether baseline familiarity was assessed before participants viewed the material (preferred) or retrospectively after viewing. If the latter, discuss potential recall and information bias.
3. **Insufficient Description of COI (Child Opportunity Index) Mapping and Categories**: Please describe how zip codes were mapped to the COI and detail the cut-offs used for “low,” “high,” and “very high.” Additionally, present the distribution of participants across COI strata, and discuss the potential ecological fallacy of using area-level COI as a proxy for individual socioeconomic status (SES).
4. **Abstract — Statistics**: Avoid stating specific p-values for numerous small comparisons in the abstract; instead, summarize the key statistically significant findings. Aim to keep the abstract concise.
5. **Definition of Abbreviations on First Use**: For instance, "COI" is used before its full name is explained in the Methods section. Ensure that all abbreviations are defined upon their first use for clarity.
6. **Consider Reporting Effect Sizes**: For key comparisons, include effect sizes (Cohen’s d, rank-biserial correlation) to better interpret clinical relevance beyond p-values.
7. **Add a Short “Implications for Practice / Next Steps” Paragraph**: Given the findings, briefly outline practical recommendations for clinicians (e.g., provider education about tissue engineering, targeted outreach to low-COI communities). However, tie these recommendations to the study limitations to ensure they remain proportional.
